# Supplementary material for: Modeling glioblastoma heterogeneity as a dynamic network of cell states
Source: Mol Syst Biol. 2021 Sep 16;17(9):e10105. doi: 10.15252/msb.202010105 (PMC8444284; doi:10.15252/msb.202010105)
Supplement: Supplementary file 6 — Source Data for Figure 5 [file MSB-17-e10105-s004.zip › Figure5A_sourcedata/GSEA_3071/nosigdb_stateII.GseaPreranked.1622795151171/NB_KI_CLUSTER5.html]

Details for gene set NB\_KI\_CLUSTER5[GSEA]

|  || Dataset | state23071 |
| Phenotype | NoPhenotypeAvailable |
| Upregulated in class | na\_pos |
| GeneSet | NB\_KI\_CLUSTER5 |
| Enrichment Score (ES) | 0.8001502 |
| Normalized Enrichment Score (NES) | 4.5888596 |
| Nominal p-value | 0.0 |
| FDR q-value | 0.0 |
| FWER p-Value | 0.0 |
Table: GSEA Results Summary

  

Fig 1: Enrichment plot: NB\_KI\_CLUSTER5      
 Profile of the Running ES Score & Positions of GeneSet Members on the Rank Ordered List

  

| PROBE | GENE SYMBOL | GENE\_TITLE | RANK IN GENE LIST | RANK METRIC SCORE | RUNNING ES | CORE ENRICHMENT || 1 | CLSPN |  |  | 1 | 1.984 | 0.0285 | Yes |
| 2 | HIST1H4C |  |  | 2 | 1.902 | 0.0560 | Yes |
| 3 | PCLAF |  |  | 10 | 1.638 | 0.0780 | Yes |
| 4 | CDK1 |  |  | 14 | 1.540 | 0.0996 | Yes |
| 5 | DIAPH3 |  |  | 15 | 1.531 | 0.1217 | Yes |
| 6 | ZWINT |  |  | 16 | 1.510 | 0.1436 | Yes |
| 7 | HIST2H2AC |  |  | 20 | 1.462 | 0.1640 | Yes |
| 8 | CENPK |  |  | 26 | 1.382 | 0.1828 | Yes |
| 9 | FBXO5 |  |  | 30 | 1.359 | 0.2017 | Yes |
| 10 | TCF19 |  |  | 32 | 1.331 | 0.2208 | Yes |
| 11 | GINS2 |  |  | 40 | 1.306 | 0.2379 | Yes |
| 12 | MCM7 |  |  | 45 | 1.273 | 0.2554 | Yes |
| 13 | HELLS |  |  | 46 | 1.259 | 0.2736 | Yes |
| 14 | BRCA1 |  |  | 49 | 1.249 | 0.2912 | Yes |
| 15 | CDT1 |  |  | 51 | 1.244 | 0.3090 | Yes |
| 16 | PCNA |  |  | 58 | 1.214 | 0.3251 | Yes |
| 17 | HIST1H1C |  |  | 60 | 1.206 | 0.3423 | Yes |
| 18 | UBE2T |  |  | 62 | 1.192 | 0.3593 | Yes |
| 19 | ATAD2 |  |  | 63 | 1.189 | 0.3765 | Yes |
| 20 | MCM5 |  |  | 74 | 1.128 | 0.3904 | Yes |
| 21 | GMNN |  |  | 83 | 1.108 | 0.4044 | Yes |
| 22 | HIST1H1E |  |  | 90 | 1.088 | 0.4187 | Yes |
| 23 | DHFR |  |  | 101 | 1.051 | 0.4314 | Yes |
| 24 | MCM3 |  |  | 105 | 1.035 | 0.4457 | Yes |
| 25 | FANCA |  |  | 109 | 1.029 | 0.4599 | Yes |
| 26 | TRIP13 |  |  | 114 | 1.014 | 0.4736 | Yes |
| 27 | DUT |  |  | 118 | 1.009 | 0.4874 | Yes |
| 28 | SMC2 |  |  | 123 | 1.005 | 0.5010 | Yes |
| 29 | CHAF1A |  |  | 127 | 0.999 | 0.5147 | Yes |
| 30 | CKS1B |  |  | 128 | 0.999 | 0.5292 | Yes |
| 31 | KIF20B |  |  | 133 | 0.989 | 0.5425 | Yes |
| 32 | RANBP1 |  |  | 149 | 0.958 | 0.5527 | Yes |
| 33 | SHCBP1 |  |  | 164 | 0.933 | 0.5627 | Yes |
| 34 | CENPM |  |  | 178 | 0.901 | 0.5725 | Yes |
| 35 | SPDL1 |  |  | 199 | 0.874 | 0.5802 | Yes |
| 36 | H2AFZ |  |  | 213 | 0.864 | 0.5895 | Yes |
| 37 | CEP152 |  |  | 217 | 0.860 | 0.6012 | Yes |
| 38 | EZH2 |  |  | 233 | 0.839 | 0.6096 | Yes |
| 39 | DBF4 |  |  | 240 | 0.832 | 0.6202 | Yes |
| 40 | RACGAP1 |  |  | 241 | 0.831 | 0.6322 | Yes |
| 41 | PRC1 |  |  | 242 | 0.830 | 0.6443 | Yes |
| 42 | CHEK1 |  |  | 252 | 0.822 | 0.6539 | Yes |
| 43 | DNMT1 |  |  | 265 | 0.792 | 0.6624 | Yes |
| 44 | TUBB4B |  |  | 271 | 0.784 | 0.6725 | Yes |
| 45 | HMGB2 |  |  | 276 | 0.780 | 0.6828 | Yes |
| 46 | SMC4 |  |  | 279 | 0.777 | 0.6936 | Yes |
| 47 | TEX30 |  |  | 287 | 0.774 | 0.7031 | Yes |
| 48 | DEK |  |  | 302 | 0.754 | 0.7105 | Yes |
| 49 | CKS2 |  |  | 312 | 0.745 | 0.7191 | Yes |
| 50 | PRR11 |  |  | 314 | 0.743 | 0.7296 | Yes |
| 51 | ANP32B |  |  | 325 | 0.734 | 0.7377 | Yes |
| 52 | CENPW |  |  | 330 | 0.727 | 0.7473 | Yes |
| 53 | LIG1 |  |  | 341 | 0.718 | 0.7552 | Yes |
| 54 | SGO2 |  |  | 415 | 0.670 | 0.7467 | Yes |
| 55 | KPNA2 |  |  | 420 | 0.669 | 0.7554 | Yes |
| 56 | RAN |  |  | 424 | 0.667 | 0.7643 | Yes |
| 57 | ZWILCH |  |  | 436 | 0.659 | 0.7711 | Yes |
| 58 | BUB3 |  |  | 441 | 0.657 | 0.7797 | Yes |
| 59 | NCAPD2 |  |  | 457 | 0.647 | 0.7853 | Yes |
| 60 | RHEB |  |  | 500 | 0.629 | 0.7840 | Yes |
| 61 | BIRC5 |  |  | 505 | 0.628 | 0.7921 | Yes |
| 62 | ECT2 |  |  | 510 | 0.626 | 0.8002 | Yes |
| 63 | MIS18BP1 |  |  | 574 | 0.588 | 0.7930 | No |
| 64 | DDX11 |  |  | 596 | 0.579 | 0.7962 | No |
| 65 | CKAP2 |  |  | 619 | 0.569 | 0.7990 | No |
| 66 | CIP2A |  |  | 696 | 0.533 | 0.7878 | No |
| 67 | NASP |  |  | 741 | 0.518 | 0.7844 | No |
| 68 | TUBA1B |  |  | 862 | 0.481 | 0.7615 | No |
| 69 | CDKN2D |  |  | 911 | 0.468 | 0.7564 | No |
| 70 | PLK1 |  |  | 1009 | 0.442 | 0.7387 | No |
| 71 | MZT1 |  |  | 1219 | 0.393 | 0.6924 | No |
| 72 | ANP32E |  |  | 1334 | 0.371 | 0.6695 | No |
| 73 | HMGB1 |  |  | 1720 | 0.308 | 0.5783 | No |
| 74 | MXD3 |  |  | 2156 | 0.256 | 0.4739 | No |
| 75 | ARL6IP1 |  |  | 3574 | -0.586 | 0.1302 | No |
Table: GSEA details [plain text format]

  

Fig 2: NB\_KI\_CLUSTER5: Random ES distribution      
 Gene set null distribution of ES for **NB\_KI\_CLUSTER5**
